# Hypoxia Impairs Initial Outgrowth of Endothelial Colony Forming Cells and Reduces Their Proliferative and Sprouting Potential

**DOI:** 10.3389/fmed.2018.00356

**Published:** 2018-12-20

**Authors:** Dimitar Tasev, Laura Dekker-Vroling, Michiel van Wijhe, Henk J. Broxterman, Pieter Koolwijk, Victor W. M. van Hinsbergh

**Affiliations:** ^1^Department of Physiology, Amsterdam Cardiovascular Sciences, Amsterdam University Medical Centers, Amsterdam, Netherlands; ^2^Department of Medical Oncology, Amsterdam University Medical Centers, Amsterdam, Netherlands

**Keywords:** ECFCs, hypoxia, colony growth, angiogenesis, tissue repair, proliferation

## Abstract

Vascular homeostasis and regeneration in ischemic tissue relies on intrinsic competence of the tissue to rapidly recruit endothelial cells for vascularization. The mononuclear cell (MNC) fraction of blood contains circulating progenitors committed to endothelial lineage. These progenitors give rise to endothelial colony-forming cells (ECFCs) that actively participate in neovascularization of ischemic tissue. To evaluate if the initial clonal outgrowth of ECFCs from cord (CB) and peripheral blood (PB) was stimulated by hypoxic conditions, MNCs obtained from CB and PB were subjected to 20 and 1% O_2_ cell culture conditions. Clonal outgrowth was followed during a 30 day incubation period. Hypoxia impaired the initial outgrowth of ECFC colonies from CB and also reduced their number that were developing from PB MNCs. Three days of oxygenation (20% O_2_) prior to hypoxia could overcome the initial CB-ECFC outgrowth. Once proliferating and subcultured the CB-ECFCs growth was only modestly affected by hypoxia; proliferation of PB-ECFCs was reduced to a similar extent (18–30% reduction). Early passages of subcultured CB- and PB-ECFCs contained only viable cells and few if any senescent cells. Tube formation by subcultured PB-ECFCs was also markedly inhibited by continuous exposure to 1% O_2_. Gene expression profiles point to regulation of the cell cycle and metabolism as major altered gene clusters. Finally we discuss our counterintuitive observations in the context of the important role that hypoxia has in promoting neovascularization.

## Introduction

The majority of wounds heal through physiological tissue repair. But if tissue repair fails, tissue engineering or regenerative medicine and transplantation is necessary (1). One of the problems during regenerative medicine is that oxygen diffusion is limited in a cellular tissue-engineered scaffold (2), resulting in reduced (hypoxia) or lack of oxygen (anoxia) within the deeper regions of the scaffold and finally cell death (2, 3). Therefore, it is important either to prevascularize tissue-engineered scaffolds by generating a blood vessel network in a scaffold *in vitro* or to create a scaffold with an environment (matrix composition, incorporation of blood vessel-generating cells and growth factors) that facilitates rapid angiogenesis when implanted in the body (4–7).

The primary vector of angiogenesis is the endothelial cell. However, in many disease conditions or after implantation of an engineered graft the ability of the endothelium to generate new vessels proceeds too slowly to overcome tissue hypoxia and subsequent cell death. As initially shown by Asahara et al. (8), within the blood the mononuclear cell (MNC) fraction expressing CD34 contains a subset of circulating progenitors committed to endothelial lineage, which proliferate at a high rate and contribute to an accelerated assembly of a new vascular network. Subsequent studies showed that the cells originally identified as endothelial progenitor cells harbored various cell types, in particular myeloid cells that acquired endothelial marker properties and endothelial colony-forming cells (ECFCs), that actively participate in neovascularization (9–13). ECFCs—also called blood-originated endothelial cells (BOECs)—exhibit high proliferative and colony-forming ability, do belong to the endothelial cell lineage and not to the hematopoietic cell lineage, and possess robust *in vitro* and *in vivo* neovascularization ability including participation in the lining of new vessels (9, 14).

Low oxygen tension in ischemic tissues determinates the fate and proliferation of progenitor or stem cells (15–17). On the one hand, hypoxia can limit growth in stem cell niches (18, 19). On the other hand, a hypoxic environment can enhance recruitment of circulating angiogenesis promoting cells, e.g., via the chemokine SDF-1 (20, 21). One may anticipate that ECFCs proliferation is also increased in hypoxic conditions, as there is a need for cells to enable expansion of the new vascular bed. However, a number of studies demonstrated that the proliferation of ECFCs was markedly inhibited by hypoxia (22–25), although some controversy exists (26, 27). Hypoxia also reduced ECFC migration as well as tubule formation into matrigel (22–25), although Decaris et al., (23) reported a difference in effect between acute and chronic hypoxia. The effect of hypoxia was mimicked by the α-ketoglutarate homolog dimethyl-oxo-glutarate (DMOG) supporting a role for HIF stabilization (24). However, the role of HIF has been debated. When the HIF-1α, one of the hypoxia-inducible factor α-subunits in endothelial cells, was overexpressed in CB-ECFCs, Kütscher et al. (28) observed improved proliferation, reduced apoptosis and increased sprouting. In contrast, recently, He et al. (21) reported that continued hypoxia reduced the proliferation of peripheral blood (PB) ECFCs by HIF-1α-mediated signaling. This differs from microvascular endothelial cells in which sprouting is enhanced by HIF-1α, while HIF-2α facilitates stabilization of vascular structures (29, 30).

In this study we summarize our findings on the effects on hypoxia on ECFCs using a custom designed hypoxia work station, which allows handing of the cells over longer periods in a defined oxygen atmosphere (30). Initially, we investigated the clonal outgrowth of ECFCs from human cord- and peripheral blood under hypoxic conditions. Subsequently, we evaluated the effect of various oxygen concentrations on the proliferation of CB- and PB-ECFCs that were cultured in the presence of platelet lysate, which improved serial propagation of ECFCs (31). Finally, we determined the effect of hypoxia on tubule formation in a fibrin matrix and compared its effect on gene expression in basal and tubule formation-stimulating conditions.

## Materials and Methods

### Isolation of CB and PB-ECFCs Under Hypoxia and Normoxia

The study was executed in accordance with the Declaration of Helsinki and was approved by the University Human Subjects Committee of the VU University Medical Center. Written informed consent was obtained from all donors in accordance with the institutional guidelines. CB-ECFCs and PB-ECFCs were isolated as previously described with minor modifications (32). Namely, after isolation of MNCs by Ficoll-Paque density gradient centrifugation, the CB- and PB-derived MNCs were re-suspended in complete EBM-2 (Lonza, Walkersville, MD, United States) supplemented with 10% FBS, 0.1% penicillin-streptomycin, 2 mM L-glutamine, and EGM-2 SingleQuotes (without hydrocortisone and gentamycin/ amphotericin-B). MNCs were divided in two equal inoculation parts and seeded in a density of at least 2.5 × 10^6^ cells per cm^2^ onto 0.1% gelatin (Sigma) pre-coated 6- or 48-well plates. One culture was placed at 20% O_2_/5% CO_2_ and the second inoculum at 1% O_2_/5% CO_2_ atmosphere.

For optimal evaluation of hypoxic conditions cells were cultured inside a custom-designed hypoxia workstation (T.C.P.S., Rotselaar, Belgium) as previously described (30).

After 3 days, the medium was renewed for the first time, followed by daily renewals during the first week; from day 7 until the end of primary culture the medium was changed every other day.

**Figure 1:**
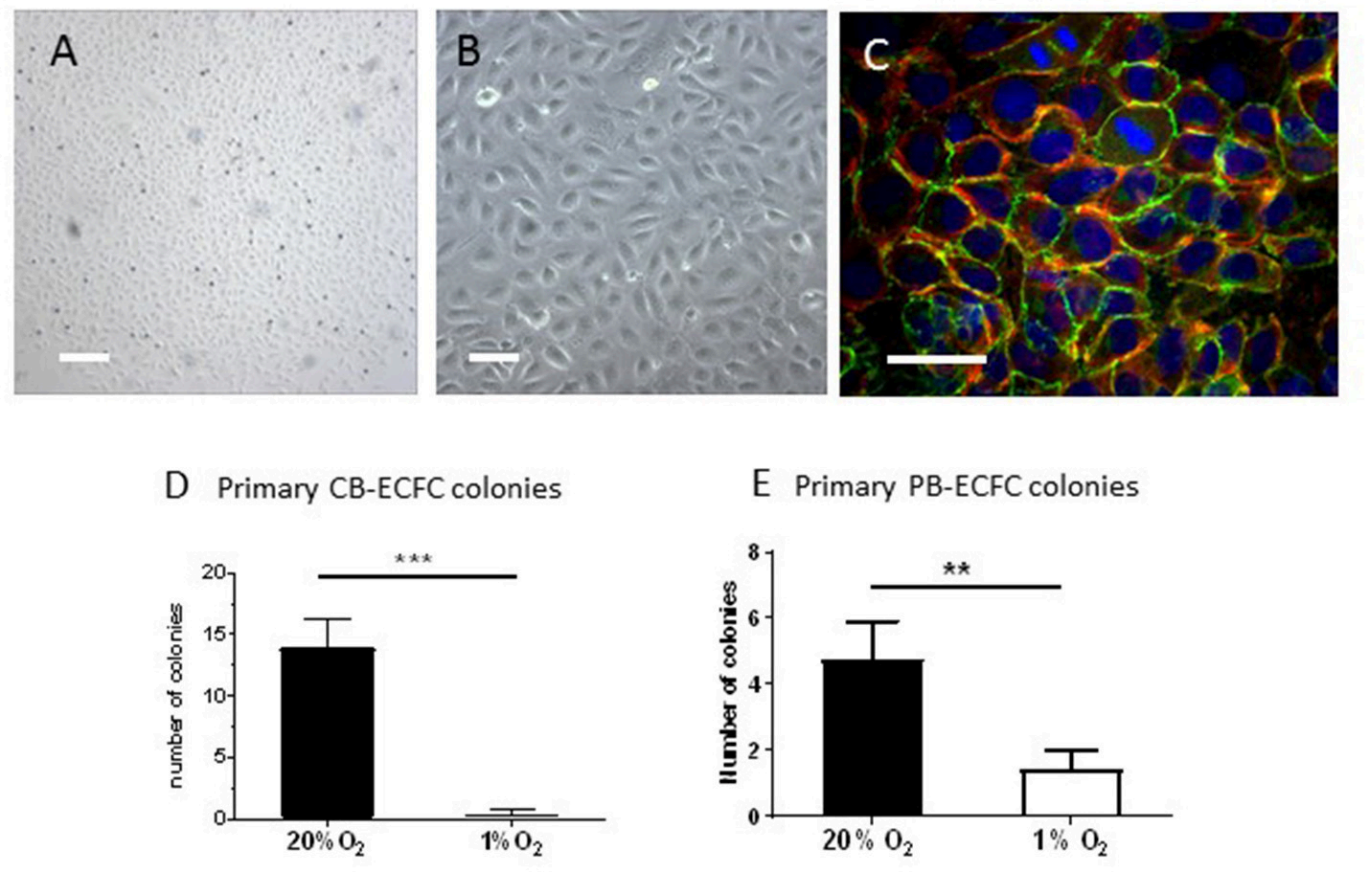
Inhibition of clonal outgrowth of ECFCs from cord and peripheral blood MNCs by hypoxia**. (A,B)** Primary colony of CB-ECFCs isolated at 1% of oxygen. Bars are 500 μm **(A)** and 100 μm **(B)**, respectively. **(C)** Staining of VE-cadherin (green), f-actin (red), and nuclei (DAPI) of a primary colony of CB-ECFCs. Note two dividing cells in the middle top part. Bar = 100 μm **(D)** Enumeration of outgrowth colonies from umbilical cord blood-derived MNCs at 20 and 1% O_2_ expressed as average number ± SEM (*n* = 14) of counted colonies per donor. Statistical significance was determined by Wilcoxon matched-pairs signed rank test; ^***^p < 0.005. **(E)** Enumeration of outgrowth colonies from peripheral blood-derived MNCs at 20 and 1% O_2_ expressed as average number ± SEM (*n* = 9) of counted colonies per donor. Statistical significance was determined by Wilcoxon matched-pairs signed rank test; ^**^p < 0.01.

The renewal medium that was used for the CB- and PB-MNCs cell cultures at 1% O_2_ was pre-incubated as a thin layer (2 ml/10 cm^2^) in empty culture dishes in the hypoxia-chamber for at least 2 h in order to allow the medium to become fully hypoxic. Renewal of cell culture medium was performed within the hypoxia-chamber preventing the cells being exposed to a normoxic environment. The outgrowth of primary ECFC colonies was monitored daily and counted in the appropriate oxygen environment by phase contrast microscopy on the basis of their characteristic endothelial cobblestone morphology (Figure 1).

Endothelial phenotype of the ECFCs obtained from primary cultures at 20 and 1% O_2_ was confirmed using immunofluorescence and flow cytometry as previously described (31). Flow cytometry showed that both CB-ECFCs and PB-ECFCs were positive for CD31, CD105, CD146, VEGFR2 (CD309), and negative for CD14, CD45, and CD133 (33, 34). Expression of CD34 was highest in dense cultures (34). In addition, the CB- and PB-ECFCs stained positive for VE-cadherin, von Willebrand factor and Acetyl-LDL uptake.

To determine the minimal exposure time to 20% O_2_ needed for overcoming the lack of colony outgrowth in 1% O_2_, CB-MNCs of three different donors were isolated and plated in individual 6-well plates which were transferred after 24, 48, 72, or 96 h (24, 48, 72, and 96h) from 20% O_2_ to 1% O_2_. Cells cultured only in 20% O_2_ or 1% O_2_ served as controls (T0). ECFC colonies were quantified when the colonies had become visible in the culture in 20% O_2_ (maximal evaluation period of 4 weeks). All experiments were performed with passage 2 (proliferation assay) to passage 5 (RNAseq experiments) CB-ECFCs or PB-ECFCs.

### Western Blot Analysis for HIF-1α and HIF-2α

Subcultured CB-ECFCs were seeded on 5 cm^2^ dishes coated with 0.1% gelatin. The dishes were placed in 1% O_2_ for various time periods (0, 3, 6, 24, 48, and 96 h). The CB-ECFCs were washed with PBS and were lysed with 100 μl Laemmli sample buffer (including β-mercaptoethanol, Biochemical, 1:20). The samples were heated for 5 min at 96°C and briefly centrifuged before loading. Equal volumes of samples were loaded for separation of HIF-1α and HIF-2α via 6% SDS-PAGE using a semi-dry transfer system (Biorad, Veenendaal, Netherlands). Rabbit polyclonal antibody HIF-1α (1:250, Cayman chemical, Ann Arbor, MI, United States) and rabbit polyclonal antibody HIF-2α (1:250, Novus Biologicals, Edinburgh, United Kingdom) and β-actin (Sigma-Aldrich, St Louis, MO, United States) were used as primary antibodies. Horse-radish peroxidase coupled anti-rabbit was used as a secondary antibody (1:250, DakoCytomation, Neverlee, Belgium).

### Transfection of CB-MNCs With dssiRNA HIF-1α and HIF-2α

Freshly isolated CB-MNCs were transfected with dssiRNA HIF-1α and HIF-2α (Qiagen, Venlo, Netherlands) using ‘Human monocyte nucleofactor kit' (Lonza, VPA-1007). Briefly, for each condition an equal amount of CB-MNCs was centrifuged, and 100 μl nucleofactor was added to the cell pellet. From both dssiRNA HIF-1α and HIF- 2α, 1 μg was added to the cell suspension, and transferred to the cuvette and placed in the electroporation system (Amaxa, Lonza Verviers) according to the manufacturer. Transfected CB-MNCs were resuspended in complete EGM medium, transferred to 0.1% gelatin coated wells, and further cultured under 1% O_2_ according the CB-ECFC culture protocol. Mock transfected cells (only electroporation step, no dssiRNA transfection) served as a control in 20 and 1% O_2_. Further details are given in Nauta et al. (30).

### Assessment of Proliferative Capacity of CB- and PB-ECFCs at 20 and 1% O_2_ Conditions

The effect of oxygen tension on proliferative capacity of sub-cultured CB- and PB-ECFCs was assessed at 20, 5, 2, and 1% O_2_ cell culture conditions. Early passages (p2–p3) of CB- and PB-ECFCs obtained from the primary colonies at 20% O_2_ cultures (*n* = 3 individual donors) were seeded at density of 500 cells/cm^2^ on 0.1% gelatin coated cell culture vehicles in complete EGM-2 and placed at 20, 5, 2, and 1% O_2_ incubators, respectively. Medium change of cultures placed at the corresponding O_2_ hypoxic chambers was performed as described in the previous section of material and methods.

Proliferation of subcultured ECFCs was calculated from cell counts. To that end, cells were incubated for indicated periods at different oxygen tensions. Subsequently they were washed, fixated with glutaraldehyde and their nuclei were stained by crystal violet. For each donor and time point/condition triplicate 10 cm^2^ wells were evaluated. From each stained well two pictures were taken at fixed positions (covering 60% of the plate surface), and the nuclei were counted using Image J software. Viability was determined by trypan blue exclusion after enzymatic detachment of unfixed cells (31). β-Galactosidase activity was assayed as previously indicated (31).

### Tube-Formation in Fibrin Matrix

Assessment of sprouting ability of PB-ECFCs expanded in PL-EGM was performed at 20% O_2_ and 1% O_2_ seeding 20,000 cells on 3D human fibrin matrices prepared as previously described (30). Following overnight incubation in M199 supplemented with 10% inactivated human serum and 10% new-born calf serum, tube formation was induced by stimulating the cells with the combination of 10 ng/ml TNF-α and 25 ng/ml VEGF_165_ and refreshed after 2 days. All growth factors were purchased from ReliaTech GmbH, Wolfenbuttel, Germany. After 96 h stimulation, the cells were fixed with 2% paraformaldehyde/HBSS and quantification of the length of formed tube-like structures was performed using Optimas image analysis software as previously described (35). The tube formation ability of PB-ECFCs was determined in triplicate wells for each of 7 donors.

### RNA Isolation and Genome-Wide RNA-Sequencing

To investigate the transcriptomic response of established PB-ECFCs under hypoxia, cells from 6 female and 6 male donors were grown at 20% O_2_ and exposed to 1% O_2_ for 24 h in EBM-2 media (SingleQuots omitted) supplemented with 5% platelet lysate prepared as previously described (31). The cell lysates were collected in 350 μL per 20 cm^2^ cells of solution containing RLT buffer (Qiagen) + 10 μL/mL β-mercaptanol. Mechanical disruption was accomplished using 1mL syringes and 21G needles, and the cell lysates were stored at −20°C overnight and transferred to −80°C the next day. Total RNA was isolated using RNeasyMinElute Cleanup Kit (Qiagen, Netherlands) and the RNA quality was tested with a Nanodrop 1,000 spectrophotometer. An RNA pool for deep sequencing of 20% O_2_ condition was prepared by mixing 2.5 μg of RNA of each donor. The same procedure was repeated for preparing the pool of 1% O_2_ RNA samples. The genome-wide RNA-sequencing was performed using the Illumina system accordingly to procedures described previously (36).

### Deep-Sequencing mRNA Analysis

Statistical analysis of genome-wide RNA-sequencing data of the biological response of PB-ECFCs during exposure to 1% O_2_ for 24 h was performed using significance analysis of microarrays (SAM) (36). The genes were defined as significantly changed by a q-value of 0.05 and an N-fold change >1.5 or < 0.66. Only genes that complied to this double criteria were further analyzed using the online tools WEB-based GEne SeT AnaLysis Toolkit Webgestalt (37) and Gene Set Enrichment Analysis (GSEA, Broad Institute, United States). For visualization of protein-protein interactions STRING10 analysis was used (38).

### Statistical Analysis

Data on initial outgrowth of colonies from cord- or peripheral blood MNC are given as Mean ± SD. Data on subcultured ECFCs are expressed as means ± SEM. At least four independent experiments, with ECFCs isolated from different donors, were performed for all analyses, unless otherwise indicated. Single comparisons were made with Student's *t* tests for normally distributed data or the Wilcoxon matched-pairs signed rank test for data not normally distributed. Comparisons between multiple groups were performed using one- or two-way ANOVA with Bonferroni *post hoc* test. Significance was defined as a *p* < 0.05.

## Results

### Hypoxia Impairs the Initial Outgrowth of ECFCs From Cord Blood—Derived Mononuclear Cell Fraction

The development of colonies of ECFCs from the MNC fraction of blood takes 10–14 days before they become visible and start to expand rapidly (also referred to as late-outgrowth EPCs). To investigate whether hypoxia stimulates the initial outgrowth of ECFCs from progenitor cells that reside within the MNC fractions of cord- and peripheral blood, CB-MNC inoculates of 14 individual donors were each incubated at 1% or 20% O_2_ (both with 5% CO_2_) for up to 30 days with regular renewal of the culture medium. The cultures were monitored daily for presence of colonies. ECFC colonies became detectable after 8–14 days and subsequently monitored and counted three times a week till the colonies started to merge. If no or only a few colonies were observed, the incubation was continued up to 30 days (to check for late coming colonies). The initial ECFC colonies were visible as densely packed cobblestone cell monolayers, which display VE-cadherin and cortical F-actin staining and multiple cell divisions (Figures 1A–C). There was no differences in morphology or differences in immunohistochemical characterization between colonies obtained at 20% or 1% of oxygen (the last one shown in Figures 1A–C).

At 20% O_2_, on average 13.9 (± 8.5) colonies were obtained from cultures of CB-MNCs (Figure 1D; individual data in Supplemental Table 3). However, under 1% O_2_ conditions 11 out of these 14 CB-MNCs generated no CB-ECFC colony outgrowth at all, while CB-ECFC outgrowth was limited to 2 colonies per isolation in the remaining 3 cultures. This failing in initiation of outgrowth resulted in a significant reduction (*p* < 0.001) of the mean number of CB-ECFCs colonies per isolation from 13.9 ± 8.5 (20% O_2_) to 0.4 ± 0.9 (1% O_2_) (Figure 1D and Supplemental Table 1).

### Initial Clonal Outgrowth of PB-ECFCs in Hypoxia

The initial outgrowth from PB-MNCs of nine individual adult donors also displayed a reduction in the number of ECFCs colonies developed in hypoxic PB-MNCs cultures as compared to their counterparts exposed to 20% oxygen levels. However, this effect was less extreme than in CB-ECFCs. Initiation of outgrowth was observed in 6 out of 9 (= 66% of total isolations) resulting in a significant (*p* < 0.05) reduction of the mean number of ECFCs colonies per isolation from 4.7 ± 3.4 (20% O_2_) to 1.4 ± 1.7 (1% O_2_) (Figure 1E and Supplemental Table 1). At the end of the colony isolation and outgrowth period of 30 days three times more colonies were counted in normoxic than in hypoxic cultures. However, those colonies that developed at 1% O_2_ also expanded rapidly, close to those that developed at 20% O_2_. Although PB-ECFCs are less sensitive to the outgrowth arrest observed with CB-ECFCs, our data indicate that hypoxia by itself is not suitable to speed up the clonal outgrowth of initial ECFC colonies.

### Effect of Oxygen Atmosphere on the Proliferation of Subcultured ECFCs

**Figure 2:**
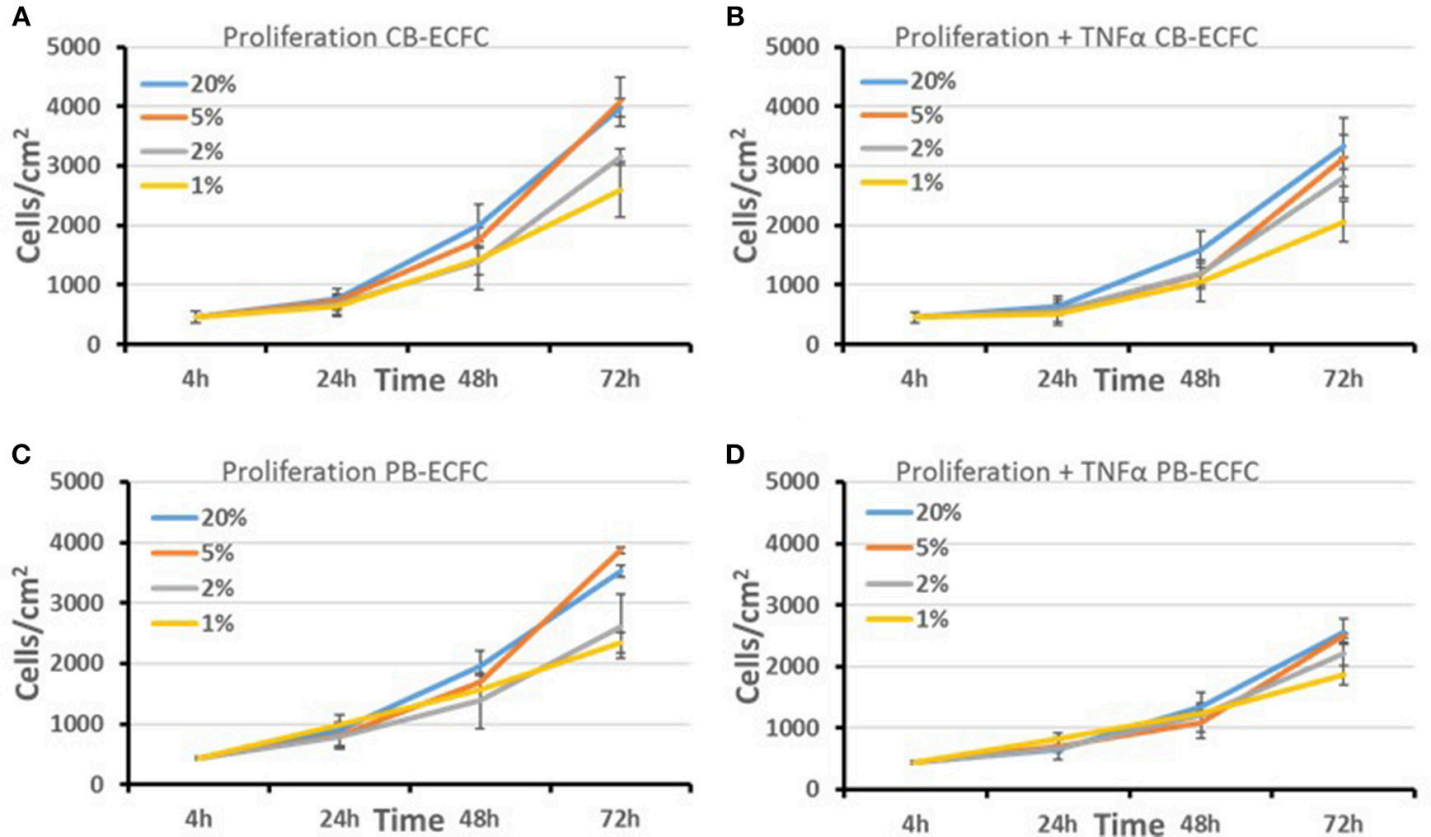
Proliferation of subcultured CB and PB-ECFCs at various oxygen concentrations. The effect of oxygen concentration (20, 5, 2, and 1%) in the absence **(A**,**C)** or presence of 10 ng/ml TNFα **(B**,**D)** on the proliferation rate of subcultured CB-ECFCs, expressed as mean number of cells/cm^2^ of 3 CB-ECFC and 3 PB-ECFC isolations of different donors ± SEM, is shown.

To evaluate whether a decrease in cell division rate could explain the lack of initial colony outgrowth we cultured already established CB-ECFCs and PB-ECFCs at low density in various oxygen atmospheres and assayed their proliferation during their logarithmic growth phase (Figures 2A,C). For each of these conditions the oxygen atmosphere (1, 2, 5, and 20%) was maintained during all manipulations, including microscopic inspection of cells and renewal of medium, which was pre-balanced at the appropriate O_2_ atmosphere (30). From the obtained proliferation data we calculated the number of divisions and cell duplication time. As a colony of 32 cells is easily detectable during initial outgrowth assay and the division time of CB-ECFCs is 1.18- to 1.30-fold faster for cells in 20% O_2_ than in 1% O_2_, a reduced proliferation can (partly) contribute to but does not explain the lack or reduction of initial colony formation by CB-ECFCs and PB-ECFCs, respectively. Furthermore, the proliferation rates of CB- and PB-ECFCs in 20 and 1% O_2_ are comparable and do not explain the fully impaired initial colony outgrowth in CB-ECFCs. Since inflammation is an important factor during angiogenesis and thereby also involved in the (out) growth of ECFCs, we evaluated the effect of the inflammatory mediator TNFα on proliferation of subcultured ECFCs. TNFα reduced the proliferation rate of ECFCs independent of the oxygen concentrations (compare Figures 2A,B, and Figures 2C,D, respectively).

### Prior Exposure to Oxygen Overcomes Hypoxia-Induced Impairment of Initial CB-ECFC Outgrowth

**Figure 3:**
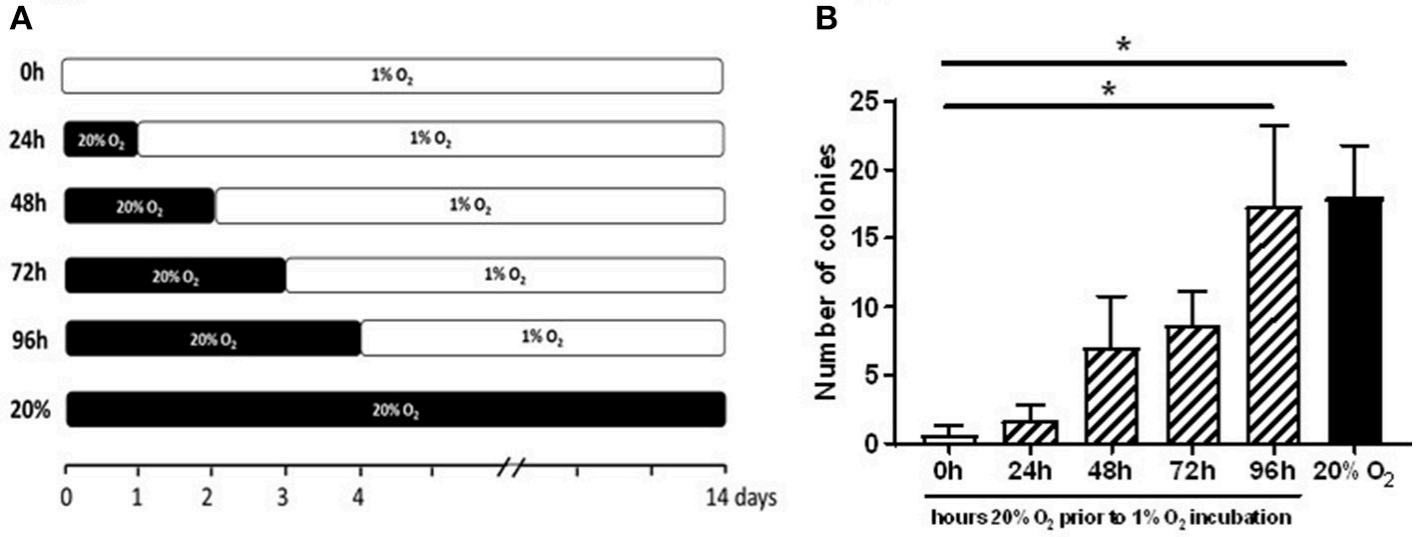
Prior exposure to 20% O_2_ restores the induction of ECFC colony formation in 1% O_2_. The graph depicts enumeration of ECFC colony outgrowth from umbilical cord blood-derived MNCs. **(A)** The freshly isolated CB-MNC fractions of three donors were seeded and exposed for varying periods (0–96 h) to ambient oxygen (20% O_2_) and subsequently transferred to 1% O_2_ atmosphere for additional culture **(B)** ECFC colony outgrowth from umbilical cord blood-derived MNCs obtained from three different donors after 24, 48, 72, or 96 h exposure to 20% O_2_. Cells cultured only in 20% O_2_ (20%), or 1% O_2_ (0 h) served as controls. ECFC colonies were quantified when the colonies had become visible in the culture in 20% O_2_ and expressed as average number ± SEM (*n* = 3) of counted colonies. Statistical significance was determined by a One-way ANOVA with Bonferroni *post-hoc* test; ^*^p < 0.05.

We hypothesized that the precursors of PB-ECFCs in the MNC fraction have been circulating in the blood, while those of CB-ECFCs may be released recently from the umbilical cord and less exposed to the oxygenated blood. Therefore, we evaluated whether temporal exposure of freshly isolated CB-MNCs to 20% oxygen contributed to the initiation of the process of ECFC colony formation by CB cells. To that end, freshly isolated CB-MNC fractions of three donors were seeded and exposed for varying periods (0–24–48–72–96 h) to ambient oxygen (20% O_2_) before transferred to 1% O_2_ atmosphere for an additional hypoxic culture up to 4 weeks. Colony formation was monitored during this 4 weeks of culturing and compared to colony generation while exposed continuously to 20% oxygen. Figure 3 shows that subsequent to 2–3 days of 20% O_2_ pre-exposure, but not shorter, initial outgrowth of ECFC colonies occurred in hypoxia. This reached statistical significance after 4 days (mean of 18 colonies in 20% of oxygen and mean of 17 colonies after 4 days of priming at 20% O_2_ and subsequent 1% O_2_ incubation). The subsequent expansion of these primed colonies proceeded fast, suggesting that the prior exposure to 20% O_2_ largely overcame the growth arrest during the initial incubation period.

In agreement with previous findings (24) and similar to in other types of endothelial cells, exposure of CB-ECFCs to hypoxia induced both HIF-1α and HIF-2α (Supplemental Image 1). Transient deletion of the combination of HIF-1α and HIF-2α residues by siRNA, which lasted for at least 72h (30), did also overcome the hypoxia-induced outgrowth arrest of ECFC colonies, similar to 20% O_2_ exposure (Supplemental Image 2, Supplemental Table 1 independent experiments, however each with low number of colonies).

### Endothelial Tube Formation by PB-ECFCs at 1 and 20% Oxygen

**Figure 4:**
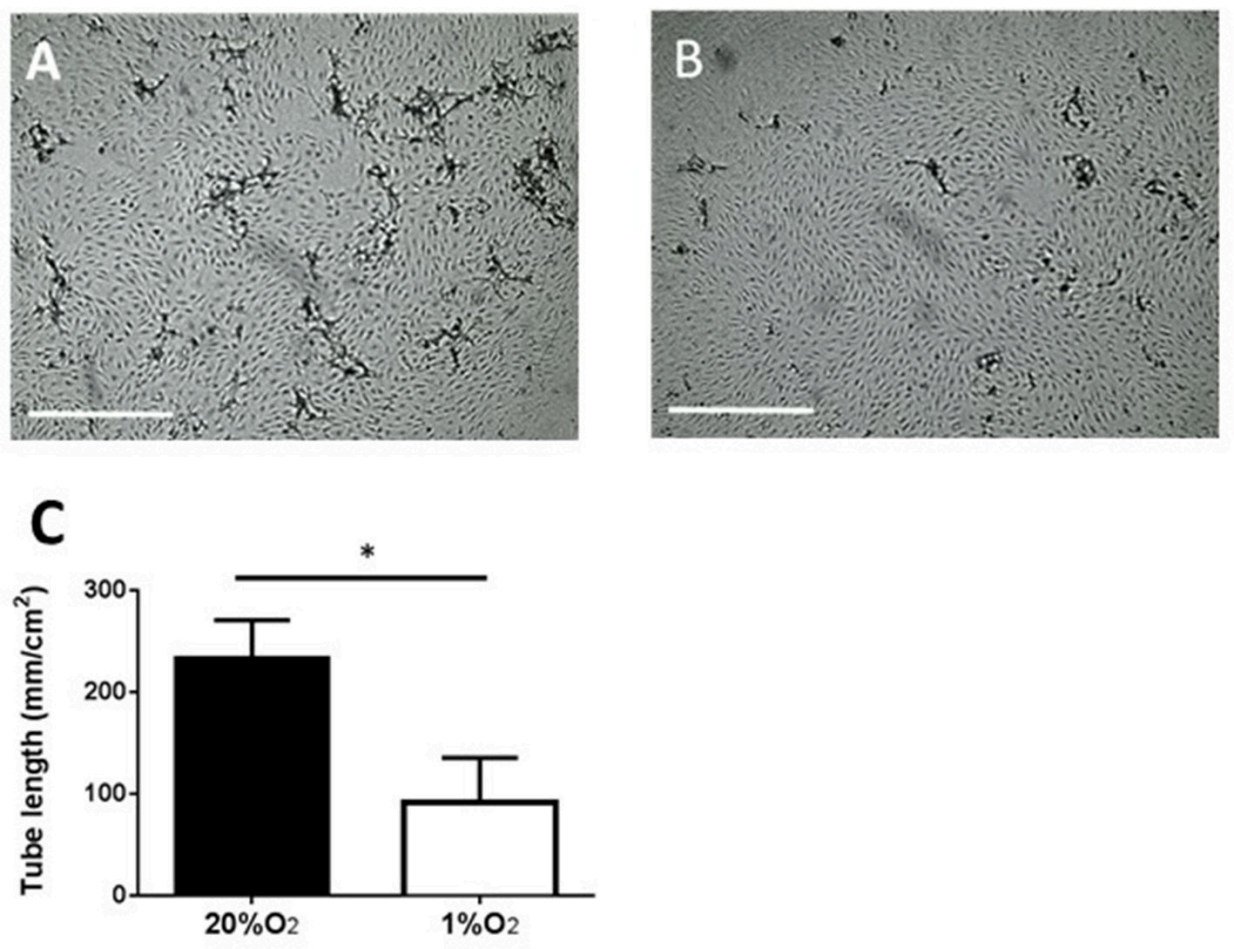
Inhibition of tube-forming capacity of PB-ECFCs when cultured at 1% O_2_. PB-ECFCs obtained from different donors were serially expanded in medium supplemented with PL at 20 or 1% of oxygen for 7 days. The PB-ECFCs were then seeded on 3D fibrin matrices and the sprouting ability of cells in fibrin matrices was then assessed after stimulation with the combination of 10 ng/ml TNFα and 25 ng/ml VEGF at 20% O_2_ (phase contrast picture **A**) or 1% O_2_ (phase contrast picture **B**) for a period of 96 h (bar = 1,000 μm). Results represent the mean ± SEM (*n* = 7) of the length of tube-like structures of the donors **(C)** Statistical significance between two oxygen concentration conditions was determined by unpaired *t*-test; ^*^p < 0.05).

Subsequently, we focused on PB-ECFCs, as their autologous nature makes them the preferential ECFC type in tissue engineering applications. For an angiogenic response endothelial cells and ECFCs not only require proliferation, but also have to form tubular structures (sprouts) that invade into the hypoxic tissue. Exposure of monolayers of PB-ECFCs on top of a 3D fibrin matrix to the combination of VEGF/TNFα indeed induced endothelial tubule invasion in the fibrin matrix when cultured in the presence of 20% O_2_. However, similar as earlier observed with human microvascular endothelial cells (30), the continuous exposure to 1% hypoxia during sprout formation significantly inhibited (59% reduction compared to PB-ECFCs at 20% O_2_, *p* < 0.05) tubule outgrowth from the PB-ECFC monolayer (Figure 4). At 20% O_2_, tubule formation in fibrin fully depends on the availability of u-PA and uPAR, which indicates that migration/invasion plays a pivotal role (33, 34). On the other hand, the effect of hypoxia on proliferation of subcultured PB-ECFCs was limited–also after TNFα-stimulation (Figure 2D), and may contribute only in a limited way to the reduction of tube formation. These effects reflect earlier findings in human microvascular endothelial cells (30).

### Gene Array Analysis of Hypoxic Response of Subcultured PB-ECFCs

**Figure 5:**
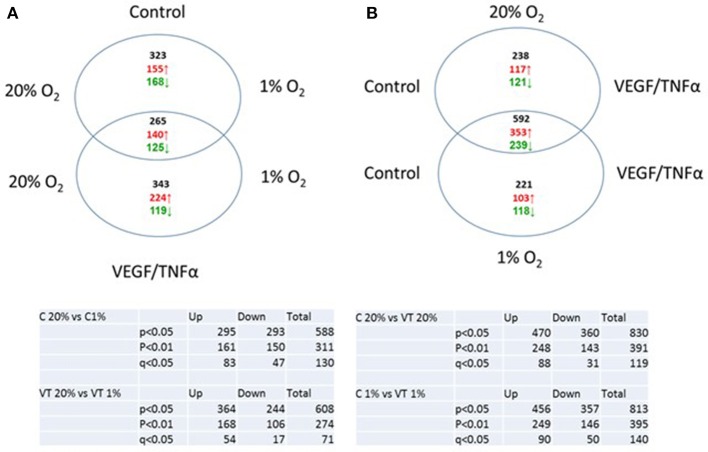
Venn diagram of genes upregulated of downregulated in control **(A)** and VEGF/TNFα-stimulated **(B)** PB-ECFCs.

**Figure 6:**
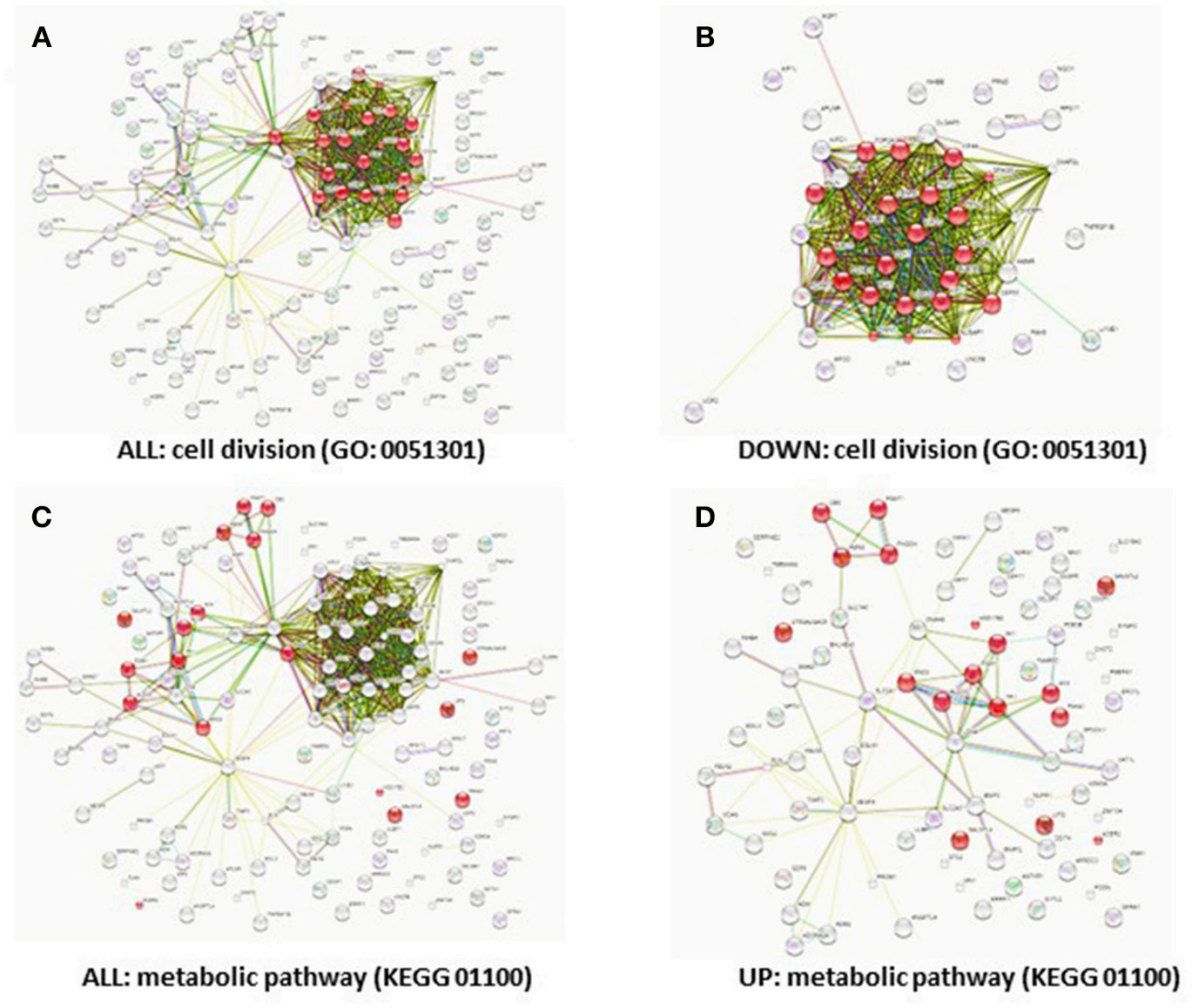
Downregulation of cell cycle genes and upregulation of metabolic genes by hypoxia. Predicted protein-protein interactions show different protein clusters. Genes that were significantly differentially regulated (FDR < 5%) in hypoxia **(A,C)**, down-regulated **(B)** or upregulated in hypoxia **(D)** were clustered based on protein-protein interactions. The nodes represent the proteins and a shared function of the proteins are shown as interconnecting lines. The genes were clustered based on the GO (0051301) cell division pathway **(A,B)** or the KEGG (01100) metabolic pathway; genes involved in these pathways are indicated in red.

While HIF-1α enhances endothelial sprouting, HIF-2α has been suggested to stabilize endothelial tubules and limits endothelial sprouting (29). We recently identified by siRNA screen four HIF-2α-regulated genes that inhibited endothelial sprouting during prolonged hypoxia: ARRDC3, MME, PPARG, and RALGPS2 (36). To evaluate whether these genes were also regulated during the response of PB-ECFCs to hypoxia, we analyzed gene expression of a pool of 10 subcultured PB-ECFCs (from 5 male and 5 female donors) that were exposed for 24 h to 20% O_2_ or 1% O_2_, both in control and in VEGF/TNFα-stimulated conditions as tubule formation was induced by the simultaneous exposure to VEGF-A and TNFα. Figure 5 summarizes the differential gene expression at the *p* < 0.05, *p* < 0.01, and *q* < 0.05 levels as revealed by genome-wide RNA-sequencing. It shows that in control and VEGF/TNFα-stimulated PB-ECFCs hypoxia altered the expression of 588 and 608 genes, respectively (*p* < 0.05). From these gene populations making up together 931 genes only 265 overlapped (140 up- and 125 down-regulated) (Figure 5). When the effects of VEGF/TNFα exposure was compared in ECFCs exposed to 1 or 20% O_2_, the overlap accounted 592 out of a total of 1041 genes (Figure 5). The top 25 up and down regulated genes (*q* < 0.05) are shown in Tables 1A-D, and all the genes (*p* < 0.05) are listed in Supplemental Data Sheets 1, 2. Pathway analysis using the online tools Gene Set Enrichment Analysis (GSEA) and the WEB-based GEne SeT AnaLysis Toolkit (Webgestalt) revealed that the upregulated genes by hypoxia showed an enrichment in several metabolic pathways (see Figure 6B) including amino acid metabolisms, glycolysis/gluconeogenesis, and carbon-, fructose- and mannose metabolism. The downregulated genes were categorized mainly in cell cycle (Figure 6B), p53 signaling pathway and cytokine-cytokine receptor interactions (Table 2). The major regulated pathways were enriched in both control and VT-treated PB-ECFCs. These data comply with anticipated roles for cell division and metabolic control in the behavior of subcultured ECFCs in hypoxia.

**Table 1A:** Top (*q* < 0.05) 25 Up regulated genes by hypoxia (non-stimulated PB-ECFCs).

**Gene symbol**	***n*-fold**	**Name**
PIK3R6	107,9	phosphoinositide-3-kinase, regulatory subunit 6
MIR210HG	22,48	MIR210 host gene (non-protein coding)
ANGPTL4	17,06	angiopoietin-like 4
GDF6	16,87	growth differentiation factor 6
SYTL2	16,32	synaptotagmin-like 2
PROM1	15,77	prominin 1
VEGFA	12,62	vascular endothelial growth factor A
ENO2	10,35	enolase 2 (gamma, neuronal)
SLC2A1	10,02	solute carrier family 2 (facilitated glucose transporter), member 1
INHBA	10	inhibin, beta A
DNAH8	9,74	dynein, axonemal, heavy chain 8
ADM2	9,19	adrenomedullin 2
FBLN2	8,85	fibulin 2
STC2	8,82	stanniocalcin 2
ELN	8,4	Elastin
GALNTL2	7,58	UDP-N-acetyl-alpha-D-galactosamine:polypeptide
		N-acetylgalactosaminyltransferase-like 2
PODN	7,11	Podocan
FER1L4	6,35	fer-1-like 4 (C. elegans) pseudogene
ALDOC	6,13	aldolase C, fructose-bisphosphate
AK4	6	adenylate kinase 4
NPTX1	5,92	neuronal pentraxin I
SDC2	5,38	syndecan 2
SMAD7	5,37	SMAD family member 7
TGFBI	5,29	transforming growth factor, beta-induced, 68 kDa
ALDH1L2	5,28	aldehyde dehydrogenase 1 family, member L2

**Table 1B:** Top (*q* < 0.05) 25 Up regulated genes by hypoxia (VT-stimulated PB-ECFCs).

**Gene symbol**	***n*-fold**	**Name**
EGLN3	61,41	egl nine homolog 3 (C. elegans)
NUPR1	13,83	nuclear protein, transcriptional regulator, 1
FABP3	12,52	fatty acid binding protein 3, muscle and heart (mammary-derived growth inhibitor)
PPARG	9,30	peroxisome proliferator-activated receptor gamma
TGFBI	9,22	transforming growth factor, beta-induced, 68kDa
DNER	7,56	delta/notch-like EGF repeat containing
SLC8A3	7,23	solute carrier family 8 (sodium/calcium exchanger), member 3
SYTL2	6,43	synaptotagmin-like 2
HIF3A	6,38	hypoxia inducible factor 3, alpha subunit
ANGPTL4	6,16	angiopoietin-like 4
COL25A1	5,05	collagen, type XXV, alpha 1
PHGDH	4,95	phosphoglycerate dehydrogenase
NDRG1	4,81	N-myc downstream regulated 1
ENO2	4,65	enolase 2 (gamma, neuronal)
ADM2	4,65	adrenomedullin 2
CSPG5	4,48	chondroitin sulfate proteoglycan 5 (neuroglycan C)
JDP2	4,39	Jun dimerization protein 2
GPNMB	4,38	glycoprotein (transmembrane) nmb
KLF4	4,28	Kruppel-like factor 4 (gut)
VEGFA	3,99	vascular endothelial growth factor A
NPTX1	3,94	neuronal pentraxin I
SLC2A1	3,88	solute carrier family 2 (facilitated glucose transporter), member 1
MEGF6	3,83	multiple EGF-like-domains 6
MN1	3,82	meningioma (disrupted in balanced translocation) 1
COL8A2	3,72	collagen, type VIII, alpha 2

**Table 1C:** Top (*q* < 0.05) 25 Down regulated genes by hypoxia (non-stimulated PB-ECFCs).

**Gene symbol**	***n*-fold**	**Name**
PRND	−22,49	prion protein 2 (dublet)
APLNR	−18,14	apelin receptor
GJA4	−15,17	gap junction protein, alpha 4, 37 kDa
INHBB	−6,37	inhibin, beta B
AQP1	−5,00	aquaporin 1 (Colton blood group)
RPS17	−4,88	ribosomal protein S17
RPS17L	−4,83	ribosomal protein S17-like
CDC20	−4,28	cell division cycle 20 homolog (S. cerevisiae)
LYVE1	−4,10	lymphatic vessel endothelial hyaluronan receptor 1
NUF2	−4,02	NUF2, NDC80 kinetochore complex component, homolog (S. cerevisiae)
HMMR	−3,74	hyaluronan-mediated motility receptor (RHAMM)
NQO1	−3,70	NAD(P)H dehydrogenase, quinone 1
UCP2	−3,63	uncoupling protein 2 (mitochondrial, proton carrier)
CKAP2L	−3,62	cytoskeleton associated protein 2-like
BUB1B	−3,54	budding uninhibited by benzimidazoles 1 homolog beta (yeast)
PAK6	−3,54	p21 protein (Cdc42/Rac)-activated kinase 6
PLK1	−3,52	polo-like kinase 1
ANLN	−3,28	anillin, actin binding protein
KIF4A	−3,28	kinesin family member 4A
SHCBP1	−3,23	SHC SH2-domain binding protein 1
BIRC5	−3,21	baculoviral IAP repeat containing 5
TPX2	−3,16	TPX2, microtubule-associated, homolog (Xenopus laevis)
SPAG5	−3,11	sperm associated antigen 5
KIFC1	−3,09	kinesin family member C1
CDCA8	−3,09	cell division cycle associated 8

**Table 1D:** Top (*q* < 0.05) 17 down regulated genes by hypoxia (VT-stimulated PB-ECFCs).

**Gene symbol**	***n*-fold**	**Name**
CCL8	−23,88	chemokine (C-C motif) ligand 8
C3	−5,96	complement component 3
IL1B	−4,88	interleukin 1, beta
KYNU	−4,51	kynureninase
EFNB2	−3,95	ephrin-B2
PLK1	−3,85	polo-like kinase 1
ADAMTS1	−3,79	ADAM metallopeptidase with thrombospondin type 1 motif, 1
DLGAP5	−3,02	discs, large (Drosophila) homolog-associated protein 5
CD200	−3,02	CD200 molecule
CHST1	−2,95	carbohydrate (keratan sulfate Gal-6) sulfotransferase 1
BUB1B	−2,66	budding uninhibited by benzimidazoles 1 homolog beta (yeast)
PAK6	−2,66	p21 protein (Cdc42/Rac)-activated kinase 6
TNFRSF11B	−2,66	tumor necrosis factor receptor superfamily, member 11b
TOP2A	−2,65	topoisomerase (DNA) II alpha 170 kDa
BMX	−2,61	BMX non-receptor tyrosine kinase
RRM2	−2,56	ribonucleotide reductase M2
MKI67	−2,44	antigen identified by monoclonal antibody Ki-67

**Table 2:** Hypoxia pathway analysis.

**Non-stimulated PB-ECFCs**			**VT-stimulated PB-ECFCs**	
**Pathway name**	**Statistics**		**Pathway name**	**Statistics**
Metabolic pathways	*p* = 3.25e−08		Metabolic pathways	*p* = 9.94e−05
Cell cycle	*p* = 1.78e−05		Cell cycle	*p* = 0.0027
Glycolysis / Gluconeogenesis	*p* = 1.78e−05		Glycolysis / Gluconeogenesis	*p* = 0.0088
Renal cell carcinoma	*p* = 0.0003		Renal cell carcinoma	*p* = 2.92e−06
Glycine, serine and threonine metabolism	*p* = 0.0003		Glycine, serine and threonine metabolism	*p* = 0.0030
TGF-beta signaling pathway	*p* = 0.0003		TGF-beta signaling pathway	*p* = 0.0112
p53 signaling pathway	*p* = 0.0031		p53 signaling pathway	*p* = 0.0088
Purine metabolism	*p* = 0.0037		Purine metabolism	*p* = 0.0052
Pentose phosphate pathway	*p* = 0.0068	
Mucin type O-Glycan biosynthesis	*p* = 0.0077	
Cysteine and methionine metabolism	*p* = 0.0093	
Fructose and mannose metabolism	*p* = 0.0093	
Cytokine-cytokine receptor interaction	*p* = 0.0140		Cytokine-cytokine receptor interaction	*p* = 4.74e−05
mTOR signaling pathway	*p* = 0.0167		
Adipocytokine signaling pathway	*p* = 0.0247		Adipocytokine signaling pathway	*p* = 0.0088
Pathways in cancer	*p* = 0.0247		Pathways in cancer	*p* = 0.0007
ECM-receptor interaction	*p* = 0.0338		
Ribosome	*p* = 0.0371		Ribosome	*p* = 0.0064
			PPAR signaling pathway	*p* = 4.74e−05
			Axon guidance	*p* = 0.0219
			Focal adhesion	*p* = 0.0450

*The KEGG pathways involved in angiogenesis and metabolism with a p < 0.05 are show. Red is upregulated and green is downregulated*.

Next, we looked at individual genes that were significantly altered by hypoxia and that were suggested earlier to contribute to endothelial sprouting. VEGF-A (as a positive regulator of angiogenesis) and ARRDC3 (negative regulator) were both significantly increased (*p* < 0.05; *q* < 0.05) in control (12.6- and 4.7-fold, respectively) and VEGF/TNFα-exposed cells (4.0- and 2.7-fold, respectively). MME and PPARG increased 2.2- and 1.9-fold in hypoxic control ECFCs (*p* < 0.05, q ns), while their mRNAs were increased by 3.2- and 9.3-fold in VEGF/TNFα-exposed ECFCs. No changes were observed in the expression of RALGPS-2. While the increase in VEGF-A may have little additional effect on cells that were already stimulated by VEGF/TNFα, changes in VEGFRs probably do. VEGFR2 decreased consistently in hypoxia-treated cells, i.e., by 50% and 51% in control and VEGF/TNFα-treated cells, respectively (*p* < 0.05, q ns). The 40% and 12% increases in VEGFR1 were not significant, but—together with the drop in VEGFR2 - are likely to decrease ECFC sprouting and/or proliferation. In control and VEGF/TNFα-exposed cells, respectively, a 20% and 16% drop in PlGF mRNA [a negative regulator as pointed out by Hookham et al. (24)] remained statistically non-significant. in VEGF/TNFα-exposed ECFCs. No changes were observed in the expression of RALGPS-2. ANGPL4, which was recently recognized as a Wnt signaling antagonist (39), HIF3A and EGLN3 (PDH-3), were strongly upregulated, particularly in VEGF/TNFα exposed ECFCs (all p and *q* < 0.05).

## Discussion

Data presented in this study indicate that hypoxia does not stimulate, but impairs the initial outgrowth of ECFC colonies from cord and peripheral blood. This inhibition is stronger in CB-ECFCs than in PB-ECFCs. In CB-ECFCs hypoxia causes an initial outgrowth arrest that could largely be overcome by a 3–4 days pre-incubation in ambient air (20% O_2_) before the explanted MNCs were transferred to the hypoxic atmosphere. Furthermore, the proliferation rate of subcultured CB- and PB-ECFCs was comparable at 5 and 20% oxygen, while it progressively dropped after exposure of the cells at 2% and 1% O_2_, independent of a proliferation reducing effect of TNFα. The ability of PB-ECFCs to form VEGF/TNFα-induced tubules in a 3D- fibrin matrix was inhibited by hypoxia. These effects were accompanied by a marked change in the expression of genes, including cell cycle, metabolism controlling, and angiogenesis controlling genes.

### Initial Colony Formation in Hypoxia

In agreement with most of these studies on CB-ECFCs, hypoxia reduced proliferation. A reduced proliferation will prolong the time period before initial colonies from explanted freshly isolated CB-MNC become visible. However, the 18–30% reduction in proliferation rate that we found with subcultured CB-ECFCs is not sufficient to explain the absence of any ECFC clone after a 30 days evaluation period as seen in 11 out of 14 cultures. Other possibilities may regard a differentiation arrest of the progenitor cells that develop into ECFC colonies or the loss of a positive interaction (or induction of a negative interaction) between hypoxic accessory MNCs and the endothelial progenitors. In preliminary experiments we could rule out the accumulation of soluble inhibitory factors as we did not find an effect of conditioned media taken from the hypoxic CB-MNC on the clonal outgrowth of subcultured CB-ECFCs. Furthermore, as 4 days pre-incubation in 20% O_2_ or transient deletion of HIF-1α and HIF-2α was sufficient to overcome the lack of colony formation it is likely that a differentiation step is needed to “awake” the rapid proliferation of CB-ECFCs. As monocyte/macrophages also express HIF-1α and HIF-2α (40), we cannot discriminate yet whether such differentiation step is within the ECFC progenitors themselves or within the accompanying mononuclear monocytes.

Several lines of research pointed to an important effect of oxygen tension on cell differentiation (41, 42). Hypoxia reversibly arrested stem cells in an undifferentiated state (42), while low oxygen tensions have also been used to maintain the pluripotency of different progenitors (43). Therefore, it is not excluded that hypoxia by maintaining a quiescence phenotype of putative endothelial progenitor cells suppresses their endothelial differentiation toward ECFCs especially if the microenvironment is devoid of pro-neovascularization clues. It is plausible that in ischemic tissues—once the pro-angiogenic environment is established—growth factors that are essential for EC differentiation such as VEFG, FGF, or HGF in conjunction with pro-angiogenic myeloid cells such as circulating angiogenic cells would eventually overcome the hypoxia-induced inhibition of differentiation of EPCs toward EC phenotype.

The initial outgrowth of PB-ECFCs was also inhibited by hypoxia, but to a much lesser extent than that of CB-ECFCs. The difference in the ability of CB-MNCs and PB-MNCs to generate primary clones under normoxia and hypoxia suggests that beside oxygen tension other factors play an important role in the initial outgrowth of ECFCs from MNCs. The microenvironment which imprint stem and progenitor cells during fetal and post-natal life can accounted for by observed differences between CB-MNCs and PB-MNCs in their ability to generate ECFCs colonies. Furthermore, to the best of our knowledge, there is no information about the circulation time of CB- and PB-endothelial progenitors. Hence, we cannot exclude that PB-endothelial progenitors have been exposed to the oxygenated milieu of the blood for a longer period than their CB counterparts. Once isolated and cultured, ECFCs obtained from umbilical cord blood differ from PB-ECFCs with respect to proliferation (43) gene expression (44–46), and *in vivo* vessel formation (46, 47).

### Hypoxia and the Propagation of Subcultured ECFCs

Hypoxia not only inhibited the initial outgrowth of ECFCs from CB- and PB-MNCs but also has an impact to the clonal and proliferative ability of subcultured cells. Interestingly, the CB-ECFCs displayed a small reduced proliferation at 1% O_2_ which is in agreement with previously reports (23, 24), while similar data have also been reported very recently on PB-ECFCs (25). The whole genome sequencing data also showed a change in metabolic pathways and in cell cycle genes during exposure to hypoxia comparable with that of human foreskin MVECs (36). While the metabolic adaptation helps the cell to overcome the limitation of energy supply, the suppression of cell cycle genes likely contributes to the hypoxia-induced reduction of ECFC proliferation. Hypoxia-driven HIF-1α activation with subsequent proliferation cell cycle arrest in G1/S phase and induction of apoptosis has been reported as a mechanism that restricts the growth of EC obtained from post-natal tissues (48). Whatever is the transcriptomic background of observed hypoxia-induced cell proliferation in CB- and PB-ECFCs, future investigation is warranted.

During neovascularization, ECFC proliferation matches the growth of the vascular tree. Our finding that hypoxia reduces ECFC proliferation is contra-intuitive suggesting that other factors that play a crucial role during *in vivo* neovascularization and are accountable for proper *in vivo* multiplication of EC are absent in our *in vitro* hypoxic assay. Indeed, rapid neovascularization reported in animal studies of addition of VEGF to ECFCs (49) as well as co-implantation of ECFCs with myeloid cells (50) or MSCs (51) pinpoint to the necessity to include these clues during *in vitro* assaying in order to unravel the true behavior of ECFCs under hypoxia.

### Endothelial Tube Formation by PB-ECFCs

Hypoxia not only reduced proliferation, but also inhibited endothelial tube formation by PB-ECFCs into a fibrin matrix. This is in line with earlier observations on CB-ECFCs (22, 24). The observed inhibition of tube formation in hypoxia was highly similar to the inhibition observed in hypoxic human microvascular EC, which was largely corrected after inhibition of HIF-2α (29, 30). Hookham et al. (24) found that Placental growth factor (PlGF) was a major player in the inhibition of tube formation by hypoxic CB-ECFCs. How PlGF exerts its effect is not yet clear. As it binds to VEGFR-1 and NRP-1 (52), it may on the one hand prevent quenching of VEGF-A by VEGFR-1 (53), and at the other hand withdraw NRP-1 from assisting cis-oriented VEGFR-2 endocytosis which is required for VEGFR-2 signaling (54). Only the latter would contribute to inhibition of tubule formation. In our experimental conditions tubule formation was fully inhibited by anti-u-PA antibodies (33) or si-uPAR (34), which suggest that migration/invasion plays a dominant role in tube formation in the fibrin matrix. A comparable mechanism has been observed in tubule formation by human microvascular endothelial cells, a process that depended on migration/invasion independent of proliferation (35).

The suppression of ECFC sprouting and proliferation by hypoxia seems contra-intuitive, but it may reflects the monolayer stabilizing properties of endothelial cells induced by HIF-2α. This response normally balances the sprouting-inducing effect of HIF-1α (29). In an hypoxic environment the surrounding non-endothelial tissue cells mainly support the HIF-1α part of this balance. However, the suggestion of a HIF-2α-suppressed ECFC sprouting contrasts to a very recent study of He et al. (25), who demonstrated by specific inhibition that inhibition of HIF-1α, but not HIF-2α, could overcome the hypoxia-induced inhibition of angiogenesis. This conclusion, which is opposite to studies on microvascular endothelial cells (29, 30), needs further evaluation and underpinning.

Altogether, there is firm consensus that hypoxia suppresses proliferation and sprouting of CB- and PB-ECFCs *in vitro*, but how the variety of suggested mediators participate and interact requires further elucidation. Notwithstanding, the crucial question raised by our findings is “why is there poor outgrowth of circulating ECFCs in hypoxia, while expansion of endothelial cells would be essential for vessel repair or new vessel forming abilities in hypoxic conditions?” One cannot exclude that this reflects the fact that ECFCs were studied in isolation, while the presence of many hypoxic tissue cells may shift the balance more into pro-angiogenic direction. Alternatively, one may anticipate that the highly proliferation of ECFCs can in particular contribute to new vessels with improved tissue circulation at the interface of the hypoxic tissue and circulating blood. If non-perfused vessel-like structures would be made in the center of a chronic hypoxic environment, it would take much energy without improvement of blood and oxygen supply. However, under these conditions, alternative sources of “endothelial precursor cells” e.g., the quiescent resident endothelial cells in vessels (55) or endothelial cells derived from erythro-myeloid progenitors (56) might be involved. Getting more insight in all these alternatives is needed to improve the use of endothelial precursor cells and ECFCs in regenerative medicine.

## Ethics Statement

The collection of cord and peripheral blood was approved and conducted according to the guidelines by the Medical Ethics Committee of the VU University medical center in Amsterdam, Netherlands.

## Author Contributions

DT prepared, performed and evaluated experiments (on PB-ECFCs) and made initial draft of the manuscript. LD-V prepared, performed and evaluated experiments (on CB-ECFCs) and made initial draft of the manuscript. MvW performed experiments. HB was involved in supervising and evaluating experiments, corrected the manuscript. PK planned and supervised experiments, evaluated and assembled data, corrected the manuscript. VvH planned and supervised, final coordination of the manuscript.

### Conflict of Interest Statement

The authors declare that the research was conducted in the absence of any commercial or financial relationships that could be construed as a potential conflict of interest.
