# Supplementary material for: Hypoxia Impairs Initial Outgrowth of Endothelial Colony Forming Cells and Reduces Their Proliferative and Sprouting Potential
Source: Front Med (Lausanne). 2018 Dec 20;5:356. doi: 10.3389/fmed.2018.00356 (PMC6306419; doi:10.3389/fmed.2018.00356)
Supplement: Supplementary file 1 [file Data_Sheet_1.PDF]

All genes regulated by hypoxia in control PB-ECFCs (q<0.05)		
**Gene symbol**	**n-fold**	**name**
ACER2	3,3	alkaline ceramidase 2
ADM	2,5	adrenomedullin
ADM2	9,2	adrenomedullin 2
ADORA2A	4,6	adenosine A2a receptor
AIF1L	-2,9	allograft inflammatory factor 1-like
AK1	2,8	adenylate kinase 1
AK4	6,0	adenylate kinase 4
ALDH1L2	5,3	aldehyde dehydrogenase 1 family, member L2
ALDOC	6,1	aldolase C, fructose-bisphosphate
ANGPTL4	17,1	angiopoietin-like 4
ANLN	-3,3	anillin, actin binding protein
ANTXR1	4,3	anthrax toxin receptor 1
APLNR	-18,1	apelin receptor
APOD	-2,8	apolipoprotein D
AQP1	-5,0	aquaporin 1 (Colton blood group)
ARRDC3	4,7	arrestin domain containing 3
ASNS	4,4	asparagine synthetase (glutamine-hydrolyzing)
BHLHE40	3,2	basic helix-loop-helix family, member e40
BIRC5	-3,2	baculoviral IAP repeat containing 5
BNIP3	2,7	BCL2/adenovirus E1B 19kDa interacting protein 3
BNIP3L	2,7	BCL2/adenovirus E1B 19kDa interacting protein 3-like
BUB1	-3,0	budding uninhibited by benzimidazoles 1 homolog (yeast)
BUB1B	-3,5	budding uninhibited by benzimidazoles 1 homolog beta (yeast)
CBS	3,6	cystathionine-beta-synthase
CCNB1	-2,8	cyclin B1
CDC20	-4,3	cell division cycle 20 homolog (S. cerevisiae)
CDCA8	-3,1	cell division cycle associated 8
CDCP1	3,7	CUB domain containing protein 1
CDH11	2,9	cadherin 11, type 2, OB-cadherin (osteoblast)
CDK1	-2,9	cyclin-dependent kinase 1
CELSR1	4,1	cadherin, EGF LAG seven-pass G-type receptor 1 (flamingo homolog, Drosophila)
CENPE	-3,0	centromere protein E, 312kDa
CENPF	-2,8	centromere protein F, 350/400kDa (mitosin)
CEP55	-3,0	centrosomal protein 55kDa
CHST2	3,2	carbohydrate (N-acetylglucosamine-6-O) sulfotransferase 2
CKAP2L	-3,6	cytoskeleton associated protein 2-like
DDIT4	3,4	DNA-damage-inducible transcript 4
DLGAP5	-3,1	discs, large (Drosophila) homolog-associated protein 5
DNAH8	9,7	dynein, axonemal, heavy chain 8
DUSP6	2,4	dual specificity phosphatase 6
EDIL3	4,3	EGF-like repeats and discoidin I-like domains 3
EGLN1	3,1	egl nine homolog 1 (C. elegans)
ELN	8,4	elastin
ENO2	10,3	enolase 2 (gamma, neuronal)
ERO1L	2,7	ERO1-like (S. cerevisiae)
ERRFI1	4,3	ERBB receptor feedback inhibitor 1
FAM65C	3,1	family with sequence similarity 65, member C
FBLN2	8,8	fibulin 2
FBLN5	4,0	fibulin 5
FER1L4	6,3	fer-1-like 4 (C. elegans) pseudogene
FOXM1	-3,0	forkhead box M1
GALNTL2	7,6	UDP-N-acetyl-alpha-D-galactosamine:polypeptide N-acetylgalactosaminyltransferase-like 2
GALNTL4	5,0	UDP-N-acetyl-alpha-D-galactosamine:polypeptide N-acetylgalactosaminyltransferase-like 4
GDF6	16,9	growth differentiation factor 6
GFRA1	2,7	GDNF family receptor alpha 1
GJA4	-15,2	gap junction protein, alpha 4, 37kDa
HMMR	-3,7	hyaluronan-mediated motility receptor (RHAMM)
HSD17B2	2,5	hydroxysteroid (17-beta) dehydrogenase 2
INHBA	10,0	inhibin, beta A
INHBB	-6,4	inhibin, beta B
JDP2	4,9	Jun dimerization protein 2
KANK1	2,4	KN motif and ankyrin repeat domains 1
KDM3A	2,5	lysine (K)-specific demethylase 3A
KIF11	-3,1	kinesin family member 11
KIF2C	-3,0	kinesin family member 2C
KIF4A	-3,3	kinesin family member 4A
KIFC1	-3,1	kinesin family member C1
KRT7	2,8	keratin 7
LDHA	3,1	lactate dehydrogenase A
LIPG	3,7	lipase, endothelial
LYVE1	-4,1	lymphatic vessel endothelial hyaluronan receptor 1
MEGF6	5,0	multiple EGF-like-domains 6
MIR210HG	22,5	MIR210 host gene (non-protein coding)
MIR4632	-2,6	microRNA 4632

MKI67	-3,0	antigen identified by monoclonal antibody Ki-67
MN1	3,1	meningioma (disrupted in balanced translocation) 1
MXI1	3,0	MAX interactor 1
MYBL2	-3,0	v-myb myeloblastosis viral oncogene homolog (avian)-like 2
NDRG1	3,9	N-myc downstream regulated 1
NPTX1	5,9	neuronal pentraxin I
NQO1	-3,7	NAD(P)H dehydrogenase, quinone 1
NUF2	-4,0	NUF2, NDC80 kinetochore complex component, homolog (S. cerevisiae)
NUPR1	3,6	nuclear protein, transcriptional regulator, 1
NUSAP1	-2,9	nucleolar and spindle associated protein 1
P4HA1	4,1	prolyl 4-hydroxylase, alpha polypeptide I
PAK6	-3,5	p21 protein (Cdc42/Rac)-activated kinase 6
PGM1	2,5	phosphoglucomutase 1
PHGDH	3,4	phosphoglycerate dehydrogenase
PIK3R6	107,9	phosphoinositide-3-kinase, regulatory subunit 6
PLK1	-3,5	polo-like kinase 1
PMEPA1	2,8	prostate transmembrane protein, androgen induced 1
PODN	7,1	podocan
PRC1	-2,8	protein regulator of cytokinesis 1
PRND	-22,5	prion protein 2 (dublet)
PROM1	15,8	prominin 1
PSAT1	4,1	phosphoserine aminotransferase 1
RPS17	-4,9	ribosomal protein S17
RPS17L	-4,8	ribosomal protein S17-like
RRM2	-3,0	ribonucleotide reductase M2
SDC2	5,4	syndecan 2
SERPINE2	2,9	serpin peptidase inhibitor, clade E (nexin, plasminogen activator inhibitor type 1), member 2
SHCBP1	-3,2	SHC SH2-domain binding protein 1
SLC16A3	3,2	solute carrier family 16, member 3 (monocarboxylic acid transporter 4)
SLC2A1	10,0	solute carrier family 2 (facilitated glucose transporter), member 1
SLC2A3	3,9	solute carrier family 2 (facilitated glucose transporter), member 3
SLC7A5	3,3	solute carrier family 7 (amino acid transporter light chain, L system), member 5
SMAD7	5,4	SMAD family member 7
SPAG5	-3,1	sperm associated antigen 5
SPOCK1	3,7	sparc/osteonectin, cwcv and kazal-like domains proteoglycan (testican) 1
ST6GALNAC6	2,8	ST6 (alpha-N-acetyl-neuraminyl-2,3-beta-galactosyl-1,3)-N-acetylgalactosaminide alpha-2,6-sialyltransferase 6
STC2	8,8	stanniocalcin 2
SYNPO	2,7	synaptopodin
SYTL2	16,3	synaptotagmin-like 2
TGFBI	5,3	transforming growth factor, beta-induced, 68kDa
TIMP3	2,7	TIMP metallopeptidase inhibitor 3
TMEM45A	4,1	transmembrane protein 45A
TNFRSF1B	-2,6	tumor necrosis factor receptor superfamily, member 1B
TOP2A	-3,0	topoisomerase (DNA) II alpha 170kDa
TPI1	2,4	triosephosphate isomerase 1
TPX2	-3,2	TPX2, microtubule-associated, homolog (Xenopus laevis)
UCP2	-3,6	uncoupling protein 2 (mitochondrial, proton carrier)
ULBP1	4,7	UL16 binding protein 1
UNC5B	-3,0	unc-5 homolog B (C. elegans)
VAT1L	4,7	vesicle amine transport protein 1 homolog (T. californica)-like
VCAN	4,5	versican
VEGFA	12,6	vascular endothelial growth factor A
VWA1	2,6	von Willebrand factor A domain containing 1
ZNF704	4,2	zinc finger protein 704
ZWINT	-2,9	ZW10 interactor
