# Supplementary material for: Hypoxia Impairs Initial Outgrowth of Endothelial Colony Forming Cells and Reduces Their Proliferative and Sprouting Potential
Source: Front Med (Lausanne). 2018 Dec 20;5:356. doi: 10.3389/fmed.2018.00356 (PMC6306419; doi:10.3389/fmed.2018.00356)
Supplement: Supplementary file 2 [file Data_Sheet_2.PDF]

All genes regulated by hypoxia in VEGF/TNFα-stimulated PB-ECFCs (q<0.05)		
**gene symbol**	**n-fold**	**Name**
ACHE	2,6	acetylcholinesterase
ADAMTS1	-3,8	ADAM metallopeptidase with thrombospondin type 1 motif, 1
ADM2	4,6	adrenomedullin 2
AK1	3,1	adenylate kinase 1
AK4	2,9	adenylate kinase 4
ALDH1L2	3,4	aldehyde dehydrogenase 1 family, member L2
ANGPTL4	6,2	angiopoietin-like 4
ANO9	2,8	anoctamin 9
ARRDC3	2,9	arrestin domain containing 3
ASNS	3,4	asparagine synthetase (glutamine-hydrolyzing)
BHLHE40	2,7	basic helix-loop-helix family, member e40
BMX	-2,6	BMX non-receptor tyrosine kinase
BUB1B	-2,7	budding uninhibited by benzimidazoles 1 homolog beta (yeast)
C15orf48	3,3	chromosome 15 open reading frame 48
C3	-6,0	complement component 3
CCL8	-23,9	chemokine (C-C motif) ligand 8
CD200	-3,0	CD200 molecule
CD82	2,4	CD82 molecule
CHST1	-2,9	carbohydrate (keratan sulfate Gal-6) sulfotransferase 1
COL25A1	5,1	collagen, type XXV, alpha 1
COL8A2	3,7	collagen, type VIII, alpha 2
CSPG5	4,5	chondroitin sulfate proteoglycan 5 (neuroglycan C)
DLGAP5	-3,0	discs, large (Drosophila) homolog-associated protein 5
DNER	7,6	delta/notch-like EGF repeat containing
EFNB2	-4,0	ephrin-B2
EGLN3	61,4	egl nine homolog 3 (C. elegans)
ENO2	4,7	enolase 2 (gamma, neuronal)
ERO1L	2,7	ERO1-like (S. cerevisiae)
ERRFI1	2,6	ERBB receptor feedback inhibitor 1
FABP3	12,5	fatty acid binding protein 3, muscle and heart (mammary-derived growth inhibitor)
GPNMB	4,4	glycoprotein (transmembrane) nmb
HIF3A	6,4	hypoxia inducible factor 3, alpha subunit
IL1B	-4,9	interleukin 1, beta
INHBA	2,7	inhibin, beta A
JDP2	4,4	Jun dimerization protein 2
KLF4	4,3	Kruppel-like factor 4 (gut)
KYNU	-4,5	kynureninase
MEGF6	3,8	multiple EGF-like-domains 6
MIR147B	3,3	microRNA 147b
MKI67	-2,4	antigen identified by monoclonal antibody Ki-67
MME	3,2	membrane metallo-endopeptidase
MN1	3,8	meningioma (disrupted in balanced translocation) 1
MTSS1	2,5	metastasis suppressor 1
NDRG1	4,8	N-myc downstream regulated 1
NPTX1	3,9	neuronal pentraxin I
NUPR1	13,8	nuclear protein, transcriptional regulator, 1
PAK6	-2,7	p21 protein (Cdc42/Rac)-activated kinase 6
PCK2	3,0	phosphoenolpyruvate carboxykinase 2 (mitochondrial)
PHGDH	5,0	phosphoglycerate dehydrogenase
PIK3IP1	3,2	phosphoinositide-3-kinase interacting protein 1
PIM1	3,2	pim-1 oncogene
PLIN2	2,4	perilipin 2
PLK1	-3,8	polo-like kinase 1
PPARG	9,3	peroxisome proliferator-activated receptor gamma
PSAT1	3,6	phosphoserine aminotransferase 1
PTGIS	3,2	prostaglandin I2 (prostacyclin) synthase
RRM2	-2,6	ribonucleotide reductase M2
SLC2A1	3,9	solute carrier family 2 (facilitated glucose transporter), member 1
SLC2A3	3,7	solute carrier family 2 (facilitated glucose transporter), member 3
SLC8A3	7,2	solute carrier family 8 (sodium/calcium exchanger), member 3
SPOCK1	2,7	sparc/osteonectin, cwcv and kazal-like domains proteoglycan (testican) 1
STC2	2,9	stanniocalcin 2
SYTL2	6,4	synaptotagmin-like 2
TGFB2	2,9	transforming growth factor, beta 2
TGFBI	9,2	transforming growth factor, beta-induced, 68kDa
TIMP3	3,0	TIMP metallopeptidase inhibitor 3
TNFRSF11B	-2,7	tumor necrosis factor receptor superfamily, member 11b
TNS1	3,3	tensin 1
TOP2A	-2,7	topoisomerase (DNA) II alpha 170kDa
TXNIP	3,4	thioredoxin interacting protein
VEGFA	4,0	vascular endothelial growth factor A
