# Supplementary figures and images for: Hypoxia Impairs Initial Outgrowth of Endothelial Colony Forming Cells and Reduces Their Proliferative and Sprouting Potential
Source: Front Med (Lausanne). 2018 Dec 20;5:356. doi: 10.3389/fmed.2018.00356 (PMC6306419; doi:10.3389/fmed.2018.00356)

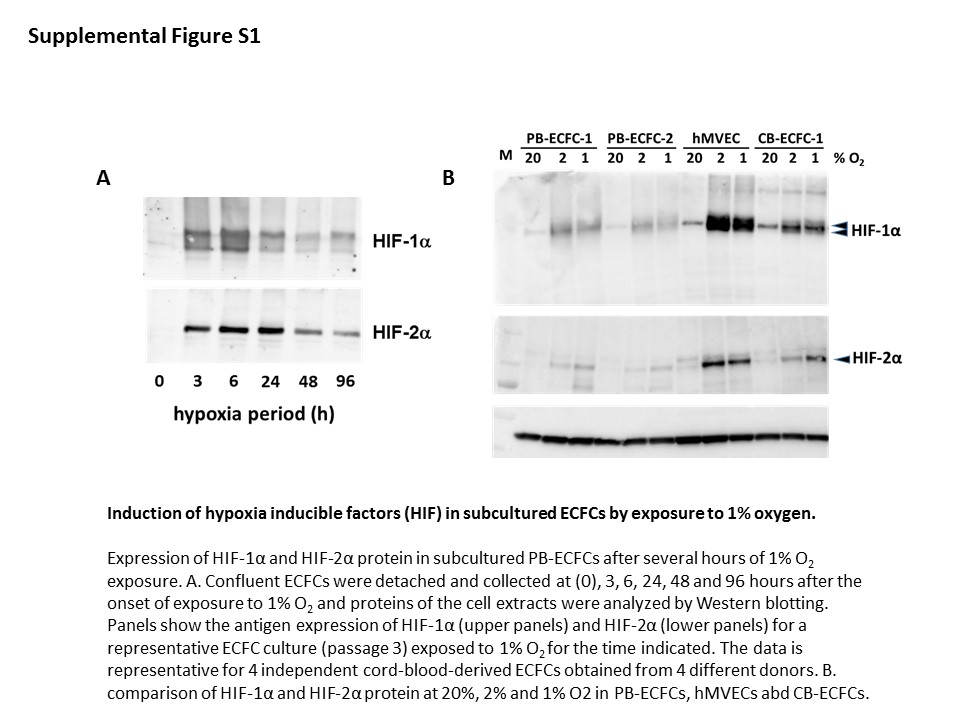

Supplement: Supplementary file 3 [file Image_1.JPEG]

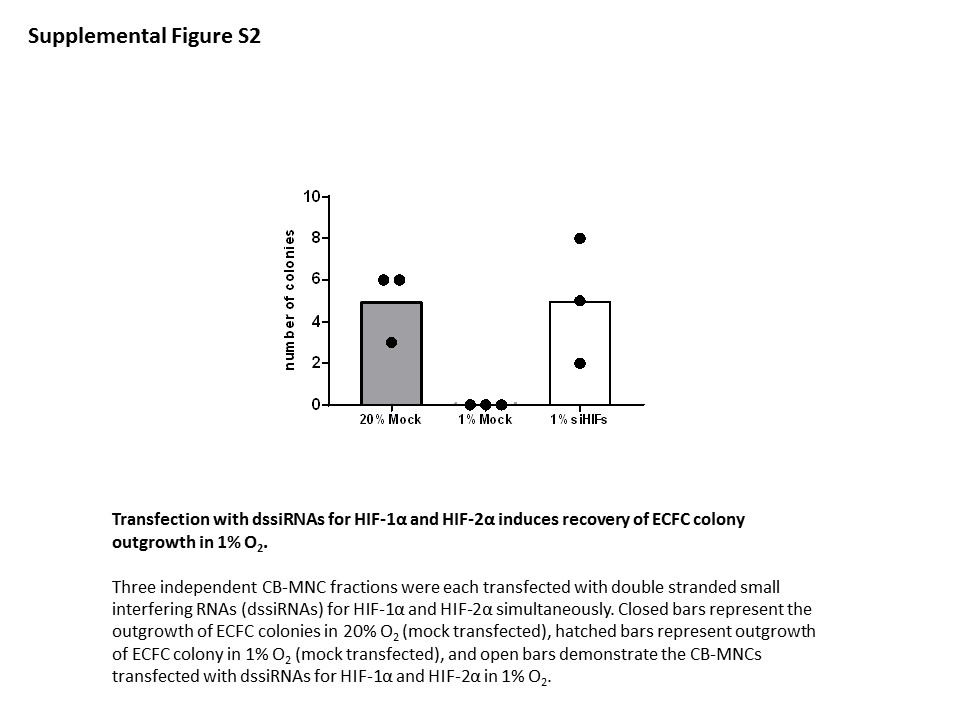

Supplement: Supplementary file 4 [file Image_2.JPEG]
