# Supplementary material for: Hypoxia Impairs Initial Outgrowth of Endothelial Colony Forming Cells and Reduces Their Proliferative and Sprouting Potential
Source: Front Med (Lausanne). 2018 Dec 20;5:356. doi: 10.3389/fmed.2018.00356 (PMC6306419; doi:10.3389/fmed.2018.00356)
Supplement: Supplementary file 5 [file Table_1.pdf]

Supplemental Table 1

Colony outgrowth from CB-MNCs per subject

Cord blood #	number of colonies	
	20% O2	1% O2
1	15	0
2	28	0
3	16	0
4	12	0
5	3	2
6	17	2
7	17	0
8	23	2
9	25	0
10	3	0
11	4	0
12	6	0
13	6	0
14	20	0
mean	13,9	0,4
std	8,5	0,9

Colony outgrowth from PB-MNCs per subject

Donor	number of colonies	
	20% O2	1% O2
PB10	7	5
PB04	6	0
PB65	12	2
PB11	3	0
PB74	3	1
PB94	6	1
PB74*	3	3
PB70	2	1
PB39	1	0
mean	4,8	1,4
std	3,4	1,7

* = donated 2x blood
